# Crystal structure of bis­[tetra­kis­(tetra­hydro­furan-κ*O*)lithium] bis[μ-2,2′,2′′-methanetriyltris(4,6-di-*tert*-butylphenolato)-κ^4^ *O*,*O*′:*O*′,*O*′′]­dimagnesiate

**DOI:** 10.1107/S2056989017008337

**Published:** 2017-06-13

**Authors:** Hongyan Zhou, Lei Wang

**Affiliations:** a College of Science, Gansu Agricultural University, Lanzhou 730070, People’s Republic of China; b College of Chemistry and Chemical Engineering, Northwest Normal University, Lanzhou 730070, People’s Republic of China

**Keywords:** crystal structure, lithium, magnesiate, heterobimetallic complex

## Abstract

The title ion-association metal complex, [Li(C_4_H_8_O)_4_]_2_[Mg_2_(C_43_H_61_O_3_)_2_], has been synthesized from the tridentate phenolic ligand tris­(3,5-di-*tert*-butyl-2-hy­droxy­phen­yl)methane in tetra­hydro­furan (THF). The aryl­oxo magnesiate complex anion is binuclear with each Mg_2_O_4_ complex unit inversion-related and bridged through the two tridentate chelating phenolate O-donors of the ligand. The complex centres have a distorted tetra­hedral stereochemistry [Mg—O range 1.8796 (17)–2.0005 (16) Å] and an Mg⋯Mg separation of 2.9430 (14) Å]. The LiO_4_ coodination sphere of the cation comprises four THF O-donor atoms and has a slightly distorted tetra­hedral conformation [Li—O range 1.899 (5)– 1.953 (5) Å]. In the crystal, a number of stabilizing intra-anion C—H⋯O hydrogen-bonding inter­actions are present but no inter-species associations are found.

## Abstract 2

The heterobimetallic complex comprises discrete Li–THF complex cations and centrosymmetric bimetallic Mg dianions with the tridentate phenolic ligand tris­(3,5-di-*tert*-butyl-2-hy­droxy­phen­yl)methane in an ion-association mode, with each metal complex core four-coordinate with distorted tetra­hedral stereochemistry.

## Chemical context   

Magnesium complexes (Wang *et al.*, 2014 ▸) and lithium complexes (Ko & Lin, 2001 ▸) display a vigorous catalytic activity in the synthesis of biodegradable polymers, through ring-opening polymerization. Heterobimetallic compounds, also called ‘ate’ complexes (Mulvey, 2009 ▸), have been systematically studied with a focus both on the elucidation of the solid-state structures and on the catalytic applications (Qiu *et al.*, 2013 ▸). We have synthesized the title metal complex, 2{[Li(THF)_4_]^+^}·[Mg_2_(C_43_H_61_O_3_)_2_]^2−^ from the reaction of the tridentate phenolic ligand tris­(3,5-di-*tert*-butyl-2-hy­droxy­phen­yl)methane with *n*-butyl-lithium and diethyl magnesium in tetra­hydro­furan (THF). The structure of this novel heterobimetallic complex, in an ion-association mode, is reported herein.
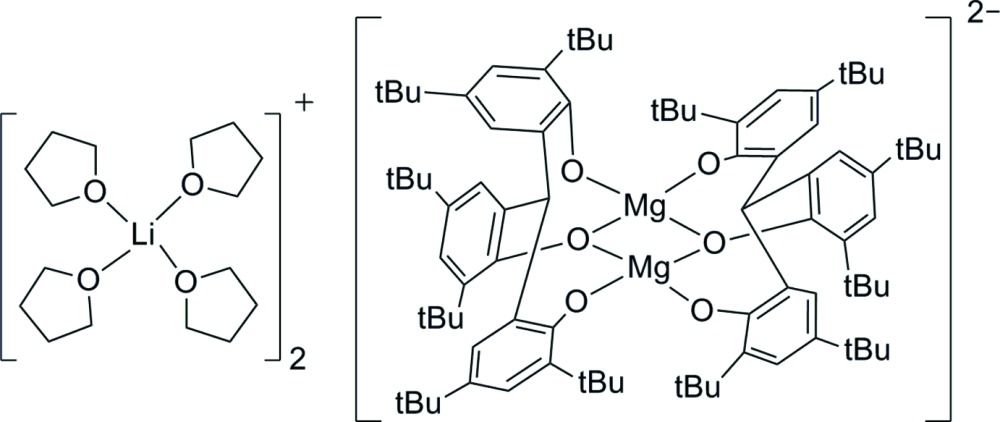


## Structural commentary   

**Figure 1:**
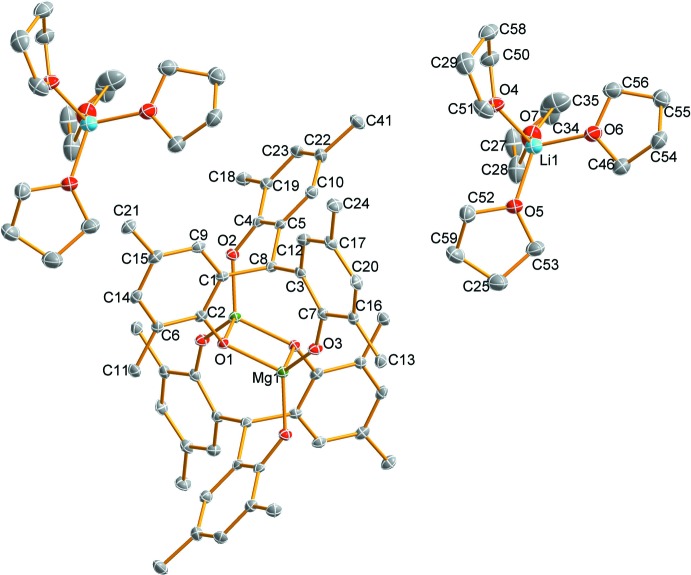
Mol­ecular structure of the title compound with displacement ellipsoids given at the 40% probability level. All of the hydrogen atoms are omitted for clarity. The non-labelled atoms of one of the two cations and the binuclear anion are generated by the symmetry operation −*x* + 1, −*y* + 2, −*z* + 1.

**Figure 2:**
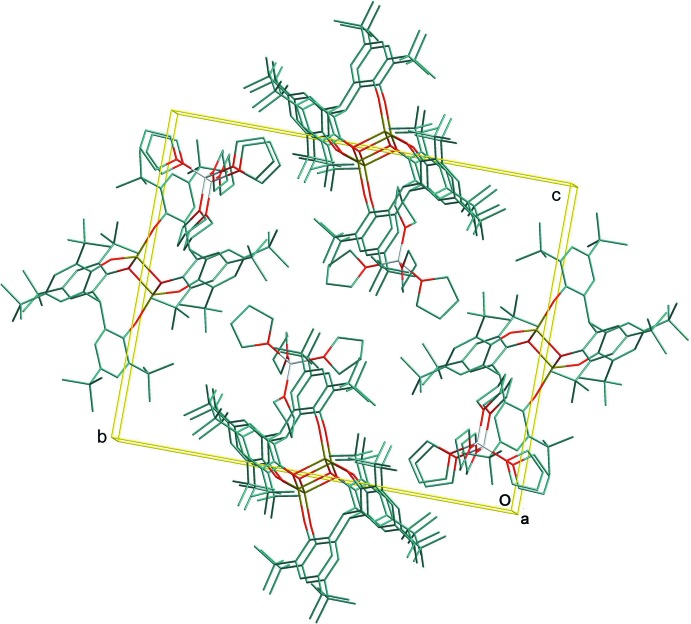
Mol­ecular packing of the title compound in the unit cell viewed along the *a* axis.

In the title complex ion-association compound (Fig. 1 ▸), the binuclear aryl­oxo magnesiate dianionic Mg_2_O_6_ complex core is centrosymmetric and is bridged through the central oxygen atoms (O1 and O1^i^) of the two chelating phenolate groups [symmetry code: (i) −*x* + 1, −*y* + 2, −*z* + 1]. The stereochemistry about each four-coordinated Mg atom is distorted tetra­hedral with Mg—O(Ar) in the range 1.8796 (17)–2.0005 (16) Å (Table 1 ▸). The dihedral angle between two planes comprising O1/Mg1/O1′ and O1/Mg1′/O1′ is 0.05 (5)°, suggesting these four atoms are almost coplanar. The Mg⋯Mg separation in the bimetallic complex is 2.9430 (14) Å. The LiO_4_ unit of the counter-ion is composed of a Li^+^ cation coordinated by four O-atom donors of the THF ligand mol­ecules, displaying a distorted tetra­hedral stereochemistry [Li—O range 1.899 (5)– 1.953 (5) Å. Within the binuclear complex anion there are six stabilizing intra-ion methyl C—H⋯O hydrogen-bonding inter­actions (Table 2 ▸), two of which are between the inversion-related ligands involving methyl group H-atom donors with a common phenolic O-atom acceptor [C42—H⋯O2^i^ and C43—H⋯O2^i^]. The absence of inter-species C—H⋯O inter­actions results in discrete cations and anions in the crystal packing (Fig. 2 ▸).

## Database survey   

A search of the Cambridge Structural Database (Groom *et al.*, 2016 ▸) revealed 39 structures of complexes having the ligand derived from tris­(3,5-di-tert-butyl-2-hy­droxy­phen­yl)methane. These include cage-like monometallic alkali complexes (Dinger & Scott, 2000 ▸) and an aluminum metal complex in an ion-association mode (Oishi *et al.*, 2016 ▸). In addition, a zinc complex based on the same ligand has been found to be useful for polymerization of cyclo­hexene oxide and carbon dioxide (Dinger & Scott, 2001 ▸).

## Synthesis and crystallization   

A solution of tris­(3,5-di-*tert*-butyl-2-hy­droxy­phen­yl)methane (0.63 g, 1.0 mmol) and ^*n*^BuLi (0.5 mL, 1.2 mmol, 2.4 *M* in hexa­ne) was stirred in THF (20 mL) at 273 K under an N_2_ atmosphere for 2 h. MgEt_2_ (1.1 mL, 1.1 mmol, 1.0 *M* in hexa­ne) was gently added to the solution. After stirring at 298 K for 6 h, the solution was filtered through celite. The filtrate was concentrated to *ca* 10 mL and cooled to 273 K to furnish colourless crystals, suitable for the X-ray analysis. Yield: 0.46 g (49%).

## Refinement   

Crystal data, data collection and structure refinement details are summarized in Table 3 ▸. Hydrogen atoms were included in the refinement at calculated positions and were allowed to ride with C—H = 0.93–0.98 Å and with *U*_iso_ = 1.2*U*_eq_(C), or 1.5*U*_eq_(methyl C).

## Supplementary Material

Crystal structure: contains datablock(s) I. DOI: 10.1107/S2056989017008337/zs2380sup1.cif

Structure factors: contains datablock(s) I. DOI: 10.1107/S2056989017008337/zs2380Isup2.hkl

CCDC reference: 1554461

Additional supporting information:  crystallographic information; 3D view; checkCIF report

## Figures and Tables

**Table 1:** Selected bond lengths (Å)

Li1—O6	1.899 (5)	Mg1—O3	1.8796 (17)
Li1—O5	1.918 (4)	Mg1—O2	1.8810 (15)
Li1—O4	1.951 (5)	Mg1—O1	1.9844 (16)
Li1—O7	1.953 (5)	Mg1—O1^i^	2.0005 (16)

Symmetry code: (i) 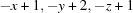.

**Table 2:** Hydrogen-bond geometry (Å, °)

*D*—H⋯*A*	*D*—H	H⋯*A*	*D*⋯*A*	*D*—H⋯*A*
C36*A*—H36*C*⋯O3	0.96	2.30	2.935 (3)	123
C39—H39*C*⋯O1	0.96	2.41	3.030 (3)	122
C40—H40*C*⋯O1	0.96	2.40	3.025 (3)	122
C42—H42*B*⋯O2^i^	0.96	2.28	2.953 (3)	126
C43—H43*B*⋯O2^i^	0.96	2.48	3.091 (3)	122
C49—H49*A*⋯O3	0.96	2.47	3.094 (3)	122

Symmetry code: (i) 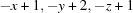.

**Table 3:** Experimental details

Crystal data	
Chemical formula	[Li(C_4_H_8_O)_4_]_2_[Mg_2_(C_43_H_61_O_3_)_2_]
*M*_r_	1891.17
Crystal system, space group	Monoclinic, *P*2_1_/*n*
Temperature (K)	293
*a*, *b*, *c* (Å)	13.6185 (4), 22.6439 (18), 18.5341 (6)
β (°)	90.811 (3)
*V* (Å^3^)	5714.9 (5)
*Z*	2
Radiation type	Mo *K*α
μ (mm^−1^)	0.08
Crystal size (mm)	0.40 × 0.21 × 0.19

Data collection	
Diffractometer	Bruker SMART 1000
No. of measured, independent and observed [*I* > 2σ(*I*)] reflections	26088, 10054, 6836
*R*_int_	0.058
(sin θ/λ)_max_ (Å^−1^)	0.595

Refinement	
*R*[*F* ^2^ > 2σ(*F* ^2^)], *wR*(*F* ^2^), *S*	0.059, 0.149, 1.05
No. of reflections	10054
No. of parameters	631
No. of restraints	1230
H-atom treatment	H-atom parameters constrained
Δρ_max_, Δρ_min_ (e Å^−3^)	0.43, −0.29

Computer programs: *SMART* and *SAINT* (Bruker, 2004 ▸), *SHELXS97*, *SHELXL97* and *SHELXTL* (Sheldrick, 2008 ▸) and *publCIF* (Westrip, 2010 ▸).

## supplementary crystallographic information

### Crystal data

**Table:**

[Li(C_4_H_8_O)_4_][Mg_2_(C_43_H_61_O_3_)_2_]	*F*(000) = 2072
*M**_r_* = 1891.17	*D*_x_ = 1.099 Mg m^−^^3^
Monoclinic, *P*2_1_/*n*	Mo *K*α radiation, λ = 0.71073 Å
Hall symbol: -P 2yn	Cell parameters from 10054 reflections
*a* = 13.6185 (4) Å	θ = 2.5–26.7°
*b* = 22.6439 (18) Å	µ = 0.08 mm^−^^1^
*c* = 18.5341 (6) Å	*T* = 293 K
β = 90.811 (3)°	Block, colorless
*V* = 5714.9 (5) Å^3^	0.40 × 0.21 × 0.19 mm
*Z* = 2	

### Data collection

**Table:**

Bruker SMART 1000 diffractometer	6836 reflections with *I* > 2σ(*I*)
Radiation source: fine-focus sealed tube	*R*_int_ = 0.058
Graphite monochromator	θ_max_ = 25.0°, θ_min_ = 3.0°
phi and ω scans	*h* = −16→16
26088 measured reflections	*k* = −26→25
10054 independent reflections	*l* = −22→22

### Refinement

**Table:**

Refinement on *F*^2^	Primary atom site location: structure-invariant direct methods
Least-squares matrix: full	Secondary atom site location: difference Fourier map
*R*[*F*^2^ > 2σ(*F*^2^)] = 0.059	Hydrogen site location: inferred from neighbouring sites
*wR*(*F*^2^) = 0.149	H-atom parameters constrained
*S* = 1.05	*w* = 1/[σ^2^(*F*_o_^2^) + (0.0528*P*)^2^ + 0.9025*P*] where *P* = (*F*_o_^2^ + 2*F*_c_^2^)/3
10054 reflections	(Δ/σ)_max_ = 0.001
631 parameters	Δρ_max_ = 0.43 e Å^−^^3^
1230 restraints	Δρ_min_ = −0.29 e Å^−^^3^

### Special details

**Table:**

Geometry. All esds (except the esd in the dihedral angle between two l.s. planes) are estimated using the full covariance matrix. The cell esds are taken into account individually in the estimation of esds in distances, angles and torsion angles; correlations between esds in cell parameters are only used when they are defined by crystal symmetry. An approximate (isotropic) treatment of cell esds is used for estimating esds involving l.s. planes.
Refinement. Refinement of F^2^ against ALL reflections. The weighted R-factor wR and goodness of fit S are based on F^2^, conventional R-factors R are based on F, with F set to zero for negative F^2^. The threshold expression of F^2^ > 2sigma(F^2^) is used only for calculating R-factors(gt) etc. and is not relevant to the choice of reflections for refinement. R-factors based on F^2^ are statistically about twice as large as those based on F, and R- factors based on ALL data will be even larger.

### Fractional atomic coordinates and isotropic or equivalent isotropic displacement parameters (Å^2^)

**Table:**

	*x*	*y*	*z*	*U*_iso_*/*U*_eq_	
C1	0.44853 (15)	0.87990 (10)	0.57566 (10)	0.0143 (5)	
C2	0.39975 (16)	0.90539 (10)	0.51568 (11)	0.0159 (5)	
C3	0.62990 (16)	0.86092 (10)	0.58242 (11)	0.0160 (5)	
C4	0.48446 (16)	0.97550 (10)	0.69623 (11)	0.0162 (5)	
C5	0.55041 (16)	0.92857 (10)	0.67975 (10)	0.0159 (5)	
C6	0.31307 (16)	0.88020 (10)	0.48672 (11)	0.0162 (5)	
C7	0.68412 (16)	0.87114 (10)	0.51879 (11)	0.0163 (5)	
C8	0.54795 (15)	0.90425 (10)	0.60218 (11)	0.0151 (5)	
H8	0.5595	0.9389	0.5717	0.018*	
C9	0.40800 (16)	0.82942 (10)	0.60705 (11)	0.0179 (5)	
H9	0.4389	0.8130	0.6474	0.022*	
C10	0.62079 (16)	0.91223 (11)	0.73083 (11)	0.0196 (5)	
H10	0.6669	0.8838	0.7185	0.024*	
C11	0.26051 (17)	0.90666 (11)	0.42000 (11)	0.0213 (5)	
C12	0.64954 (17)	0.81067 (11)	0.62311 (11)	0.0198 (5)	
H12	0.6101	0.8029	0.6625	0.024*	
C13	0.83216 (16)	0.84588 (11)	0.43869 (12)	0.0206 (5)	
C14	0.27818 (16)	0.82944 (10)	0.52025 (11)	0.0186 (5)	
H14	0.2213	0.8121	0.5016	0.022*	
C15	0.32317 (17)	0.80296 (10)	0.58002 (11)	0.0198 (5)	
C16	0.76638 (16)	0.83422 (10)	0.50446 (11)	0.0183 (5)	
C17	0.72462 (17)	0.77159 (11)	0.60822 (11)	0.0207 (5)	
C18	0.41599 (17)	1.05073 (11)	0.78728 (11)	0.0225 (6)	
C19	0.48607 (16)	0.99987 (10)	0.76694 (11)	0.0184 (5)	
C20	0.78393 (16)	0.78616 (11)	0.54998 (11)	0.0209 (5)	
H20	0.8382	0.7625	0.5410	0.025*	
C21	0.27898 (18)	0.74571 (11)	0.61067 (12)	0.0229 (5)	
C22	0.62541 (16)	0.93650 (11)	0.79964 (12)	0.0228 (6)	
C23	0.55636 (17)	0.97928 (11)	0.81606 (12)	0.0222 (5)	
H23	0.5571	0.9951	0.8624	0.027*	
C24	0.74578 (18)	0.71642 (11)	0.65421 (12)	0.0254 (6)	
C25	0.5483 (2)	0.57398 (15)	0.10178 (15)	0.0507 (9)	
H25A	0.5606	0.6024	0.0638	0.061*	
H25B	0.5452	0.5348	0.0808	0.061*	
C26	0.8408 (2)	0.72517 (15)	0.69722 (16)	0.0547 (9)	
H26A	0.8543	0.6905	0.7254	0.082*	
H26B	0.8341	0.7586	0.7287	0.082*	
H26C	0.8938	0.7320	0.6647	0.082*	
C27	0.5095 (3)	0.44057 (17)	0.3707 (2)	0.0841 (13)	
H27A	0.4457	0.4496	0.3906	0.101*	
H27B	0.5076	0.4013	0.3498	0.101*	
C28	0.5386 (2)	0.48648 (15)	0.31469 (18)	0.0539 (9)	
H28A	0.5856	0.4703	0.2811	0.065*	
H28B	0.4816	0.5007	0.2879	0.065*	
C29	0.5060 (2)	0.76988 (14)	0.40393 (15)	0.0487 (8)	
H29A	0.5357	0.8088	0.4045	0.058*	
H29B	0.4351	0.7740	0.4022	0.058*	
C30	0.7192 (3)	0.85111 (16)	0.85394 (17)	0.0687 (11)	
H30A	0.7708	0.8405	0.8873	0.103*	
H30B	0.7354	0.8375	0.8065	0.103*	
H30C	0.6588	0.8332	0.8686	0.103*	
C31	0.6866 (2)	0.93678 (18)	0.93004 (14)	0.0644 (11)	
H31A	0.7387	0.9232	0.9613	0.097*	
H31B	0.6256	0.9198	0.9449	0.097*	
H31C	0.6824	0.9790	0.9327	0.097*	
C32	0.8032 (2)	0.94579 (19)	0.82828 (18)	0.0698 (12)	
H32A	0.7976	0.9880	0.8293	0.105*	
H32B	0.8167	0.9331	0.7800	0.105*	
H32C	0.8557	0.9336	0.8600	0.105*	
C33	0.7558 (3)	0.66158 (13)	0.60625 (16)	0.0566 (9)	
H33A	0.8119	0.6660	0.5759	0.085*	
H33B	0.6977	0.6572	0.5768	0.085*	
H33C	0.7640	0.6272	0.6361	0.085*	
C34	0.5872 (3)	0.44511 (17)	0.4264 (2)	0.0826 (12)	
H34A	0.6401	0.4177	0.4165	0.099*	
H34B	0.5613	0.4365	0.4737	0.099*	
C35	0.6222 (3)	0.50667 (15)	0.42268 (17)	0.0626 (10)	
H35A	0.6005	0.5287	0.4644	0.075*	
H35B	0.6934	0.5076	0.4218	0.075*	
C36A	0.86788 (18)	0.90987 (11)	0.43636 (13)	0.0279 (6)	
H36A	0.9112	0.9149	0.3964	0.042*	
H36B	0.9023	0.9191	0.4805	0.042*	
H36C	0.8125	0.9358	0.4307	0.042*	
C37	0.17009 (18)	0.87032 (12)	0.39790 (13)	0.0315 (6)	
H37A	0.1250	0.8691	0.4372	0.047*	
H37B	0.1900	0.8309	0.3860	0.047*	
H37C	0.1387	0.8882	0.3567	0.047*	
C38	0.92361 (17)	0.80626 (12)	0.43914 (13)	0.0303 (6)	
H38A	0.9628	0.8152	0.3979	0.045*	
H38B	0.9038	0.7656	0.4374	0.045*	
H38C	0.9614	0.8132	0.4824	0.045*	
C39	0.22469 (19)	0.96932 (12)	0.43508 (13)	0.0309 (6)	
H39A	0.1820	0.9689	0.4759	0.046*	
H39B	0.1895	0.9840	0.3936	0.046*	
H39C	0.2800	0.9944	0.4452	0.046*	
C40	0.32951 (18)	0.90733 (12)	0.35515 (11)	0.0288 (6)	
H40A	0.2946	0.9216	0.3133	0.043*	
H40B	0.3528	0.8680	0.3462	0.043*	
H40C	0.3843	0.9328	0.3654	0.043*	
C41	0.70742 (18)	0.91836 (13)	0.85285 (12)	0.0318 (6)	
C42	0.30853 (18)	1.03454 (12)	0.77105 (13)	0.0305 (6)	
H42A	0.2898	1.0017	0.8006	0.046*	
H42B	0.3013	1.0240	0.7211	0.046*	
H42C	0.2673	1.0678	0.7813	0.046*	
C43	0.44292 (19)	1.10648 (11)	0.74497 (13)	0.0289 (6)	
H43A	0.4003	1.1382	0.7585	0.043*	
H43B	0.4355	1.0990	0.6942	0.043*	
H43C	0.5098	1.1171	0.7557	0.043*	
C44I	0.2683 (2)	0.69991 (12)	0.54984 (14)	0.0363 (7)	
H44A	0.3315	0.6922	0.5296	0.054*	
H44B	0.2248	0.7150	0.5130	0.054*	
H44C	0.2419	0.6640	0.5690	0.054*	
C45	0.17617 (19)	0.75797 (12)	0.64056 (14)	0.0369 (7)	
H45A	0.1481	0.7218	0.6577	0.055*	
H45B	0.1350	0.7741	0.6030	0.055*	
H45C	0.1813	0.7857	0.6797	0.055*	
C46	0.82584 (19)	0.63296 (13)	0.28622 (13)	0.0348 (7)	
H46A	0.8015	0.6598	0.2494	0.042*	
H46B	0.8432	0.5959	0.2635	0.042*	
C47	0.3430 (2)	0.71873 (12)	0.67013 (14)	0.0372 (7)	
H47A	0.4069	0.7102	0.6516	0.056*	
H47B	0.3133	0.6829	0.6868	0.056*	
H47C	0.3490	0.7461	0.7095	0.056*	
C48	0.4233 (2)	1.06616 (13)	0.86792 (13)	0.0409 (7)	
H48A	0.4885	1.0798	0.8793	0.061*	
H48B	0.4092	1.0317	0.8961	0.061*	
H48C	0.3768	1.0966	0.8788	0.061*	
C49	0.77385 (19)	0.83204 (13)	0.36928 (12)	0.0340 (7)	
H49A	0.7155	0.8558	0.3674	0.051*	
H49B	0.7560	0.7910	0.3688	0.051*	
H49C	0.8135	0.8406	0.3282	0.051*	
C50	0.5239 (2)	0.67180 (13)	0.44191 (13)	0.0375 (7)	
H50A	0.5682	0.6451	0.4671	0.045*	
H50B	0.4569	0.6590	0.4498	0.045*	
C51	0.5422 (2)	0.73363 (12)	0.34117 (14)	0.0411 (7)	
H51A	0.4982	0.7376	0.2999	0.049*	
H51B	0.6073	0.7464	0.3273	0.049*	
C52	0.48956 (19)	0.63168 (13)	0.19563 (14)	0.0364 (7)	
H52A	0.4942	0.6710	0.1752	0.044*	
H52B	0.4452	0.6328	0.2361	0.044*	
C53	0.6279 (2)	0.57714 (13)	0.15967 (13)	0.0387 (7)	
H53A	0.6464	0.5378	0.1757	0.046*	
H53B	0.6858	0.5969	0.1416	0.046*	
C54	0.91390 (19)	0.65918 (13)	0.32510 (13)	0.0382 (7)	
H54A	0.9117	0.7020	0.3246	0.046*	
H54B	0.9748	0.6460	0.3039	0.046*	
C55	0.9026 (2)	0.63515 (14)	0.40088 (14)	0.0399 (7)	
H55A	0.9274	0.5951	0.4047	0.048*	
H55B	0.9363	0.6598	0.4361	0.048*	
C56	0.7932 (2)	0.63710 (14)	0.41053 (13)	0.0420 (7)	
H56A	0.7729	0.6082	0.4459	0.050*	
H56B	0.7726	0.6760	0.4262	0.050*	
C57	0.6634 (2)	0.70310 (14)	0.70703 (16)	0.0491 (8)	
H57A	0.6778	0.6671	0.7323	0.074*	
H57B	0.6024	0.6990	0.6809	0.074*	
H57C	0.6582	0.7349	0.7410	0.074*	
C58	0.5385 (2)	0.73428 (13)	0.46798 (14)	0.0416 (7)	
H58A	0.4984	0.7426	0.5096	0.050*	
H58B	0.6069	0.7418	0.4802	0.050*	
C59	0.4546 (2)	0.58831 (14)	0.13988 (14)	0.0439 (8)	
H59A	0.4065	0.6059	0.1073	0.053*	
H59B	0.4264	0.5535	0.1620	0.053*	
Li1	0.6174 (3)	0.6098 (2)	0.3193 (2)	0.0323 (10)	
Mg1	0.56849 (5)	0.97310 (3)	0.44884 (4)	0.01547 (18)	
O1	0.43576 (10)	0.95620 (7)	0.48655 (7)	0.0154 (3)	
O2	0.57524 (11)	1.00281 (7)	0.35426 (7)	0.0184 (4)	
O3	0.65886 (11)	0.91317 (7)	0.47250 (7)	0.0196 (4)	
O4	0.54464 (13)	0.67304 (8)	0.36606 (8)	0.0329 (4)	
O5	0.58526 (12)	0.61056 (8)	0.21824 (8)	0.0330 (5)	
O6	0.75222 (13)	0.62355 (9)	0.34078 (9)	0.0417 (5)	
O7	0.58148 (15)	0.53233 (9)	0.35698 (10)	0.0454 (5)	

### Atomic displacement parameters (Å^2^)

**Table:**

	*U*^11^	*U*^22^	*U*^33^	*U*^12^	*U*^13^	*U*^23^
C1	0.0177 (12)	0.0123 (12)	0.0129 (10)	0.0016 (10)	0.0023 (9)	−0.0013 (9)
C2	0.0188 (12)	0.0125 (12)	0.0165 (11)	−0.0014 (10)	0.0055 (9)	−0.0031 (9)
C3	0.0156 (11)	0.0181 (13)	0.0142 (11)	−0.0009 (10)	−0.0022 (9)	−0.0022 (9)
C4	0.0177 (12)	0.0147 (13)	0.0163 (11)	−0.0035 (10)	0.0029 (9)	0.0040 (9)
C5	0.0163 (11)	0.0187 (13)	0.0129 (11)	−0.0052 (10)	0.0023 (9)	0.0021 (9)
C6	0.0154 (12)	0.0174 (13)	0.0156 (11)	0.0001 (10)	0.0015 (9)	−0.0041 (9)
C7	0.0165 (12)	0.0149 (13)	0.0173 (11)	−0.0012 (10)	−0.0021 (9)	−0.0007 (9)
C8	0.0158 (11)	0.0148 (12)	0.0147 (11)	−0.0005 (10)	0.0018 (9)	0.0028 (9)
C9	0.0212 (12)	0.0167 (13)	0.0160 (11)	0.0033 (10)	0.0048 (9)	0.0005 (9)
C10	0.0191 (12)	0.0218 (14)	0.0180 (11)	0.0011 (11)	0.0027 (9)	0.0003 (10)
C11	0.0216 (13)	0.0231 (14)	0.0192 (12)	−0.0039 (11)	−0.0037 (10)	−0.0010 (10)
C12	0.0222 (12)	0.0200 (13)	0.0174 (11)	0.0010 (11)	0.0005 (9)	0.0013 (10)
C13	0.0156 (12)	0.0236 (14)	0.0226 (12)	0.0013 (11)	0.0017 (9)	−0.0006 (10)
C14	0.0178 (12)	0.0175 (13)	0.0206 (12)	−0.0057 (10)	0.0041 (9)	−0.0033 (10)
C15	0.0240 (13)	0.0136 (13)	0.0219 (12)	−0.0008 (11)	0.0084 (10)	−0.0012 (10)
C16	0.0146 (12)	0.0203 (13)	0.0200 (11)	−0.0007 (10)	−0.0022 (9)	−0.0031 (10)
C17	0.0218 (12)	0.0183 (13)	0.0220 (12)	0.0014 (11)	−0.0040 (10)	−0.0002 (10)
C18	0.0256 (13)	0.0229 (14)	0.0191 (12)	0.0023 (11)	0.0035 (10)	−0.0038 (10)
C19	0.0186 (12)	0.0180 (13)	0.0186 (11)	−0.0026 (10)	0.0041 (9)	−0.0002 (10)
C20	0.0160 (12)	0.0199 (14)	0.0268 (12)	0.0066 (10)	−0.0023 (10)	−0.0016 (10)
C21	0.0274 (13)	0.0151 (13)	0.0265 (13)	−0.0030 (11)	0.0073 (10)	0.0015 (10)
C22	0.0178 (12)	0.0292 (15)	0.0213 (12)	−0.0025 (11)	−0.0015 (10)	−0.0001 (11)
C23	0.0236 (13)	0.0278 (15)	0.0151 (11)	−0.0028 (11)	−0.0002 (10)	−0.0054 (10)
C24	0.0291 (14)	0.0205 (14)	0.0265 (13)	0.0027 (12)	−0.0037 (11)	0.0075 (11)
C25	0.060 (2)	0.055 (2)	0.0366 (16)	0.0127 (18)	−0.0061 (15)	−0.0125 (15)
C26	0.054 (2)	0.045 (2)	0.064 (2)	0.0029 (17)	−0.0238 (17)	0.0214 (16)
C27	0.054 (2)	0.046 (2)	0.152 (4)	−0.014 (2)	−0.010 (2)	0.041 (2)
C28	0.0352 (18)	0.041 (2)	0.086 (2)	0.0002 (16)	−0.0012 (16)	0.0051 (18)
C29	0.0508 (19)	0.0290 (18)	0.066 (2)	0.0086 (15)	−0.0055 (16)	−0.0081 (15)
C30	0.092 (3)	0.060 (3)	0.053 (2)	0.025 (2)	−0.0426 (19)	−0.0009 (18)
C31	0.059 (2)	0.105 (3)	0.0287 (16)	0.035 (2)	−0.0208 (15)	−0.0112 (17)
C32	0.0282 (17)	0.117 (4)	0.063 (2)	−0.008 (2)	−0.0183 (16)	0.018 (2)
C33	0.094 (3)	0.0279 (18)	0.0485 (18)	0.0159 (18)	0.0055 (18)	0.0082 (15)
C34	0.076 (3)	0.051 (3)	0.120 (3)	0.001 (2)	−0.001 (2)	0.031 (2)
C35	0.091 (3)	0.050 (2)	0.0477 (19)	0.003 (2)	0.0140 (18)	0.0165 (16)
C36A	0.0258 (14)	0.0279 (16)	0.0302 (13)	0.0017 (12)	0.0078 (11)	0.0027 (11)
C37	0.0283 (14)	0.0401 (18)	0.0260 (13)	−0.0109 (13)	−0.0071 (11)	0.0007 (12)
C38	0.0234 (14)	0.0280 (16)	0.0398 (15)	0.0029 (12)	0.0098 (11)	−0.0011 (12)
C39	0.0284 (14)	0.0271 (16)	0.0369 (14)	0.0056 (12)	−0.0113 (12)	0.0017 (12)
C40	0.0315 (15)	0.0369 (17)	0.0179 (12)	−0.0066 (13)	−0.0009 (11)	0.0002 (11)
C41	0.0262 (14)	0.0467 (19)	0.0222 (13)	0.0050 (13)	−0.0070 (11)	−0.0023 (12)
C42	0.0265 (14)	0.0312 (16)	0.0342 (14)	0.0035 (12)	0.0126 (11)	−0.0012 (12)
C43	0.0321 (15)	0.0232 (15)	0.0316 (14)	0.0022 (12)	0.0038 (11)	−0.0053 (11)
C44I	0.0452 (17)	0.0204 (15)	0.0435 (16)	−0.0130 (13)	0.0122 (13)	−0.0027 (12)
C45	0.0376 (16)	0.0282 (16)	0.0452 (16)	−0.0104 (13)	0.0158 (13)	0.0049 (13)
C46	0.0369 (16)	0.0327 (17)	0.0349 (15)	−0.0003 (14)	0.0078 (12)	−0.0018 (12)
C47	0.0511 (18)	0.0188 (15)	0.0419 (15)	−0.0072 (14)	0.0047 (13)	0.0091 (12)
C48	0.0540 (19)	0.0411 (19)	0.0277 (14)	0.0174 (15)	0.0047 (13)	−0.0102 (13)
C49	0.0291 (15)	0.0468 (19)	0.0261 (13)	0.0027 (14)	0.0053 (11)	−0.0078 (12)
C50	0.0405 (17)	0.0394 (18)	0.0329 (15)	−0.0023 (14)	0.0084 (12)	−0.0017 (13)
C51	0.0541 (19)	0.0289 (17)	0.0399 (16)	0.0014 (15)	−0.0140 (14)	0.0050 (13)
C52	0.0290 (15)	0.0391 (18)	0.0412 (16)	−0.0013 (14)	0.0015 (12)	−0.0039 (13)
C53	0.0445 (17)	0.0407 (18)	0.0310 (14)	0.0056 (15)	0.0044 (13)	−0.0082 (13)
C54	0.0321 (15)	0.0406 (19)	0.0421 (16)	0.0041 (14)	0.0096 (12)	−0.0037 (13)
C55	0.0415 (17)	0.0377 (18)	0.0403 (16)	0.0074 (15)	−0.0056 (13)	−0.0035 (13)
C56	0.0466 (18)	0.050 (2)	0.0297 (15)	−0.0036 (16)	0.0037 (13)	−0.0040 (13)
C57	0.054 (2)	0.0378 (19)	0.0558 (19)	0.0061 (16)	0.0092 (15)	0.0231 (15)
C58	0.0430 (17)	0.0408 (19)	0.0413 (16)	−0.0056 (15)	0.0112 (13)	−0.0141 (14)
C59	0.0417 (17)	0.053 (2)	0.0364 (16)	−0.0059 (16)	−0.0067 (13)	−0.0076 (14)
Li1	0.038 (3)	0.031 (3)	0.028 (2)	0.002 (2)	0.0028 (19)	−0.0008 (19)
Mg1	0.0166 (4)	0.0146 (4)	0.0152 (4)	0.0004 (3)	0.0006 (3)	0.0011 (3)
O1	0.0165 (8)	0.0134 (9)	0.0163 (7)	−0.0017 (7)	−0.0011 (6)	0.0025 (6)
O2	0.0194 (8)	0.0188 (9)	0.0169 (8)	0.0036 (7)	−0.0003 (6)	0.0032 (7)
O3	0.0216 (9)	0.0193 (9)	0.0179 (8)	0.0060 (7)	0.0027 (6)	0.0046 (7)
O4	0.0433 (11)	0.0266 (11)	0.0288 (9)	0.0030 (9)	0.0064 (8)	−0.0029 (8)
O5	0.0347 (11)	0.0374 (12)	0.0270 (9)	0.0024 (9)	0.0014 (8)	−0.0064 (8)
O6	0.0349 (11)	0.0592 (15)	0.0312 (10)	−0.0059 (10)	0.0063 (8)	−0.0072 (9)
O7	0.0496 (13)	0.0292 (12)	0.0576 (12)	−0.0003 (10)	0.0047 (10)	0.0115 (10)

### Geometric parameters (Å, º)

**Table:**

Li1—O6	1.899 (5)	C31—H31C	0.9600
Li1—O5	1.918 (4)	C32—C41	1.520 (4)
Li1—O4	1.951 (5)	C32—H32A	0.9600
Li1—O7	1.953 (5)	C32—H32B	0.9600
Mg1—O3	1.8796 (17)	C32—H32C	0.9600
Mg1—O2	1.8810 (15)	C33—H33A	0.9600
Mg1—O1	1.9844 (16)	C33—H33B	0.9600
Mg1—O1^i^	2.0005 (16)	C33—H33C	0.9600
C1—C9	1.400 (3)	C34—C35	1.475 (5)
C1—C2	1.410 (3)	C34—H34A	0.9700
C1—C8	1.536 (3)	C34—H34B	0.9700
C2—O1	1.365 (3)	C35—O7	1.452 (4)
C2—C6	1.410 (3)	C35—H35A	0.9700
C3—C12	1.389 (3)	C35—H35B	0.9700
C3—C7	1.419 (3)	C36A—H36A	0.9600
C3—C8	1.534 (3)	C36A—H36B	0.9600
C4—O2^i^	1.325 (2)	C36A—H36C	0.9600
C4—C19	1.422 (3)	C37—H37A	0.9600
C4—C5	1.427 (3)	C37—H37B	0.9600
C5—C10	1.388 (3)	C37—H37C	0.9600
C5—C8	1.540 (3)	C38—H38A	0.9600
C6—C14	1.393 (3)	C38—H38B	0.9600
C6—C11	1.541 (3)	C38—H38C	0.9600
C7—O3	1.324 (3)	C39—H39A	0.9600
C7—C16	1.426 (3)	C39—H39B	0.9600
C8—H8	0.9800	C39—H39C	0.9600
C9—C15	1.389 (3)	C40—H40A	0.9600
C9—H9	0.9300	C40—H40B	0.9600
C10—C22	1.389 (3)	C40—H40C	0.9600
C10—H10	0.9300	C42—H42A	0.9600
C11—C39	1.527 (3)	C42—H42B	0.9600
C11—C37	1.532 (3)	C42—H42C	0.9600
C11—C40	1.536 (3)	C43—H43A	0.9600
C12—C17	1.383 (3)	C43—H43B	0.9600
C12—H12	0.9300	C43—H43C	0.9600
C13—C36A	1.529 (3)	C44I—H44A	0.9600
C13—C49	1.535 (3)	C44I—H44B	0.9600
C13—C38	1.535 (3)	C44I—H44C	0.9600
C13—C16	1.546 (3)	C45—H45A	0.9600
C14—C15	1.394 (3)	C45—H45B	0.9600
C14—H14	0.9300	C45—H45C	0.9600
C15—C21	1.541 (3)	C46—O6	1.450 (3)
C16—C20	1.396 (3)	C46—C54	1.512 (4)
C17—C20	1.397 (3)	C46—H46A	0.9700
C17—C24	1.537 (3)	C46—H46B	0.9700
C18—C43	1.533 (3)	C47—H47A	0.9600
C18—C42	1.534 (3)	C47—H47B	0.9600
C18—C48	1.537 (3)	C47—H47C	0.9600
C18—C19	1.546 (3)	C48—H48A	0.9600
C19—C23	1.392 (3)	C48—H48B	0.9600
C20—H20	0.9300	C48—H48C	0.9600
C21—C47	1.523 (3)	C49—H49A	0.9600
C21—C44I	1.537 (3)	C49—H49B	0.9600
C21—C45	1.538 (3)	C49—H49C	0.9600
C22—C23	1.387 (3)	C50—O4	1.438 (3)
C22—C41	1.535 (3)	C50—C58	1.507 (4)
C23—H23	0.9300	C50—H50A	0.9700
C24—C26	1.523 (4)	C50—H50B	0.9700
C24—C57	1.530 (4)	C51—O4	1.448 (3)
C24—C33	1.534 (4)	C51—H51A	0.9700
C25—C59	1.503 (4)	C51—H51B	0.9700
C25—C53	1.516 (4)	C52—O5	1.445 (3)
C25—H25A	0.9700	C52—C59	1.499 (4)
C25—H25B	0.9700	C52—H52A	0.9700
C26—H26A	0.9600	C52—H52B	0.9700
C26—H26B	0.9600	C53—O5	1.451 (3)
C26—H26C	0.9600	C53—H53A	0.9700
C27—C34	1.471 (5)	C53—H53B	0.9700
C27—C28	1.526 (4)	C54—C55	1.516 (4)
C27—H27A	0.9700	C54—H54A	0.9700
C27—H27B	0.9700	C54—H54B	0.9700
C28—O7	1.421 (4)	C55—C56	1.503 (4)
C28—H28A	0.9700	C55—H55A	0.9700
C28—H28B	0.9700	C55—H55B	0.9700
C29—C58	1.497 (4)	C56—O6	1.434 (3)
C29—C51	1.512 (4)	C56—H56A	0.9700
C29—H29A	0.9700	C56—H56B	0.9700
C29—H29B	0.9700	C57—H57A	0.9600
C30—C41	1.531 (4)	C57—H57B	0.9600
C30—H30A	0.9600	C57—H57C	0.9600
C30—H30B	0.9600	C58—H58A	0.9700
C30—H30C	0.9600	C58—H58B	0.9700
C31—C41	1.521 (3)	C59—H59A	0.9700
C31—H31A	0.9600	C59—H59B	0.9700
C31—H31B	0.9600	O2—C4^i^	1.325 (2)
			
O6—Li1—O5	114.2 (2)	C27—C34—H34A	110.7
O6—Li1—O4	106.4 (2)	C35—C34—H34A	110.7
O5—Li1—O4	108.5 (2)	C27—C34—H34B	110.7
O6—Li1—O7	108.6 (2)	C35—C34—H34B	110.7
O5—Li1—O7	107.6 (2)	H34A—C34—H34B	108.8
O4—Li1—O7	111.7 (2)	O7—C35—C34	107.3 (3)
O3—Mg1—O2	115.80 (7)	O7—C35—H35A	110.3
O3—Mg1—O1	112.04 (7)	C34—C35—H35A	110.3
O2—Mg1—O1	116.99 (7)	O7—C35—H35B	110.3
O3—Mg1—O1^i^	117.55 (7)	C34—C35—H35B	110.3
O2—Mg1—O1^i^	105.89 (7)	H35A—C35—H35B	108.5
O1—Mg1—O1^i^	84.79 (7)	C13—C36A—H36A	109.5
Mg1—O1—Mg1^i^	95.21 (7)	C13—C36A—H36B	109.5
C4^i^—O2—Mg1	138.86 (14)	H36A—C36A—H36B	109.5
C7—O3—Mg1	146.48 (14)	C13—C36A—H36C	109.5
C9—C1—C2	118.5 (2)	H36A—C36A—H36C	109.5
C9—C1—C8	120.74 (19)	H36B—C36A—H36C	109.5
C2—C1—C8	120.56 (19)	C11—C37—H37A	109.5
O1—C2—C6	119.60 (19)	C11—C37—H37B	109.5
O1—C2—C1	119.27 (19)	H37A—C37—H37B	109.5
C6—C2—C1	121.1 (2)	C11—C37—H37C	109.5
C12—C3—C7	119.1 (2)	H37A—C37—H37C	109.5
C12—C3—C8	122.0 (2)	H37B—C37—H37C	109.5
C7—C3—C8	118.87 (19)	C13—C38—H38A	109.5
O2^i^—C4—C19	120.6 (2)	C13—C38—H38B	109.5
O2^i^—C4—C5	120.42 (18)	H38A—C38—H38B	109.5
C19—C4—C5	118.99 (19)	C13—C38—H38C	109.5
C10—C5—C4	118.89 (19)	H38A—C38—H38C	109.5
C10—C5—C8	123.2 (2)	H38B—C38—H38C	109.5
C4—C5—C8	117.44 (18)	C11—C39—H39A	109.5
C14—C6—C2	116.9 (2)	C11—C39—H39B	109.5
C14—C6—C11	121.4 (2)	H39A—C39—H39B	109.5
C2—C6—C11	121.7 (2)	C11—C39—H39C	109.5
O3—C7—C3	121.5 (2)	H39A—C39—H39C	109.5
O3—C7—C16	119.9 (2)	H39B—C39—H39C	109.5
C3—C7—C16	118.6 (2)	C11—C40—H40A	109.5
C3—C8—C1	109.57 (18)	C11—C40—H40B	109.5
C3—C8—C5	116.45 (17)	H40A—C40—H40B	109.5
C1—C8—C5	115.76 (17)	C11—C40—H40C	109.5
C3—C8—H8	104.5	H40A—C40—H40C	109.5
C1—C8—H8	104.5	H40B—C40—H40C	109.5
C5—C8—H8	104.5	C32—C41—C31	110.0 (3)
C15—C9—C1	122.2 (2)	C32—C41—C30	108.7 (3)
C15—C9—H9	118.9	C31—C41—C30	106.3 (2)
C1—C9—H9	118.9	C32—C41—C22	108.5 (2)
C5—C10—C22	122.9 (2)	C31—C41—C22	112.8 (2)
C5—C10—H10	118.6	C30—C41—C22	110.5 (2)
C22—C10—H10	118.6	C18—C42—H42A	109.5
C39—C11—C37	106.9 (2)	C18—C42—H42B	109.5
C39—C11—C40	109.5 (2)	H42A—C42—H42B	109.5
C37—C11—C40	107.12 (18)	C18—C42—H42C	109.5
C39—C11—C6	111.12 (18)	H42A—C42—H42C	109.5
C37—C11—C6	111.6 (2)	H42B—C42—H42C	109.5
C40—C11—C6	110.45 (19)	C18—C43—H43A	109.5
C17—C12—C3	123.6 (2)	C18—C43—H43B	109.5
C17—C12—H12	118.2	H43A—C43—H43B	109.5
C3—C12—H12	118.2	C18—C43—H43C	109.5
C36A—C13—C49	109.3 (2)	H43A—C43—H43C	109.5
C36A—C13—C38	107.19 (19)	H43B—C43—H43C	109.5
C49—C13—C38	107.18 (19)	C21—C44I—H44A	109.5
C36A—C13—C16	111.86 (19)	C21—C44I—H44B	109.5
C49—C13—C16	109.11 (18)	H44A—C44I—H44B	109.5
C38—C13—C16	112.03 (19)	C21—C44I—H44C	109.5
C6—C14—C15	124.1 (2)	H44A—C44I—H44C	109.5
C6—C14—H14	118.0	H44B—C44I—H44C	109.5
C15—C14—H14	118.0	C21—C45—H45A	109.5
C9—C15—C14	117.1 (2)	C21—C45—H45B	109.5
C9—C15—C21	123.9 (2)	H45A—C45—H45B	109.5
C14—C15—C21	119.0 (2)	C21—C45—H45C	109.5
C20—C16—C7	118.2 (2)	H45A—C45—H45C	109.5
C20—C16—C13	120.9 (2)	H45B—C45—H45C	109.5
C7—C16—C13	120.8 (2)	O6—C46—C54	106.08 (19)
C12—C17—C20	116.1 (2)	O6—C46—H46A	110.5
C12—C17—C24	122.9 (2)	C54—C46—H46A	110.5
C20—C17—C24	121.0 (2)	O6—C46—H46B	110.5
C43—C18—C42	109.3 (2)	C54—C46—H46B	110.5
C43—C18—C48	107.3 (2)	H46A—C46—H46B	108.7
C42—C18—C48	107.1 (2)	C21—C47—H47A	109.5
C43—C18—C19	109.62 (19)	C21—C47—H47B	109.5
C42—C18—C19	111.4 (2)	H47A—C47—H47B	109.5
C48—C18—C19	112.0 (2)	C21—C47—H47C	109.5
C23—C19—C4	118.3 (2)	H47A—C47—H47C	109.5
C23—C19—C18	120.75 (19)	H47B—C47—H47C	109.5
C4—C19—C18	120.82 (19)	C18—C48—H48A	109.5
C16—C20—C17	123.7 (2)	C18—C48—H48B	109.5
C16—C20—H20	118.1	H48A—C48—H48B	109.5
C17—C20—H20	118.1	C18—C48—H48C	109.5
C47—C21—C44I	107.9 (2)	H48A—C48—H48C	109.5
C47—C21—C45	109.0 (2)	H48B—C48—H48C	109.5
C44I—C21—C45	108.0 (2)	C13—C49—H49A	109.5
C47—C21—C15	112.4 (2)	C13—C49—H49B	109.5
C44I—C21—C15	109.31 (19)	H49A—C49—H49B	109.5
C45—C21—C15	110.1 (2)	C13—C49—H49C	109.5
C23—C22—C10	117.1 (2)	H49A—C49—H49C	109.5
C23—C22—C41	122.4 (2)	H49B—C49—H49C	109.5
C10—C22—C41	120.5 (2)	O4—C50—C58	105.6 (2)
C22—C23—C19	123.5 (2)	O4—C50—H50A	110.6
C22—C23—H23	118.2	C58—C50—H50A	110.6
C19—C23—H23	118.2	O4—C50—H50B	110.6
C26—C24—C57	108.4 (2)	C58—C50—H50B	110.6
C26—C24—C33	109.1 (2)	H50A—C50—H50B	108.8
C57—C24—C33	106.5 (2)	O4—C51—C29	106.0 (2)
C26—C24—C17	109.6 (2)	O4—C51—H51A	110.5
C57—C24—C17	112.4 (2)	C29—C51—H51A	110.5
C33—C24—C17	110.7 (2)	O4—C51—H51B	110.5
C59—C25—C53	105.1 (2)	C29—C51—H51B	110.5
C59—C25—H25A	110.7	H51A—C51—H51B	108.7
C53—C25—H25A	110.7	O5—C52—C59	105.1 (2)
C59—C25—H25B	110.7	O5—C52—H52A	110.7
C53—C25—H25B	110.7	C59—C52—H52A	110.7
H25A—C25—H25B	108.8	O5—C52—H52B	110.7
C24—C26—H26A	109.5	C59—C52—H52B	110.7
C24—C26—H26B	109.5	H52A—C52—H52B	108.8
H26A—C26—H26B	109.5	O5—C53—C25	105.3 (2)
C24—C26—H26C	109.5	O5—C53—H53A	110.7
H26A—C26—H26C	109.5	C25—C53—H53A	110.7
H26B—C26—H26C	109.5	O5—C53—H53B	110.7
C34—C27—C28	103.8 (3)	C25—C53—H53B	110.7
C34—C27—H27A	111.0	H53A—C53—H53B	108.8
C28—C27—H27A	111.0	C46—C54—C55	102.1 (2)
C34—C27—H27B	111.0	C46—C54—H54A	111.3
C28—C27—H27B	111.0	C55—C54—H54A	111.3
H27A—C27—H27B	109.0	C46—C54—H54B	111.3
O7—C28—C27	103.4 (3)	C55—C54—H54B	111.3
O7—C28—H28A	111.1	H54A—C54—H54B	109.2
C27—C28—H28A	111.1	C56—C55—C54	102.3 (2)
O7—C28—H28B	111.1	C56—C55—H55A	111.3
C27—C28—H28B	111.1	C54—C55—H55A	111.3
H28A—C28—H28B	109.1	C56—C55—H55B	111.3
C58—C29—C51	102.8 (2)	C54—C55—H55B	111.3
C58—C29—H29A	111.2	H55A—C55—H55B	109.2
C51—C29—H29A	111.2	O6—C56—C55	105.1 (2)
C58—C29—H29B	111.2	O6—C56—H56A	110.7
C51—C29—H29B	111.2	C55—C56—H56A	110.7
H29A—C29—H29B	109.1	O6—C56—H56B	110.7
C41—C30—H30A	109.5	C55—C56—H56B	110.7
C41—C30—H30B	109.5	H56A—C56—H56B	108.8
H30A—C30—H30B	109.5	C24—C57—H57A	109.5
C41—C30—H30C	109.5	C24—C57—H57B	109.5
H30A—C30—H30C	109.5	H57A—C57—H57B	109.5
H30B—C30—H30C	109.5	C24—C57—H57C	109.5
C41—C31—H31A	109.5	H57A—C57—H57C	109.5
C41—C31—H31B	109.5	H57B—C57—H57C	109.5
H31A—C31—H31B	109.5	C29—C58—C50	102.4 (2)
C41—C31—H31C	109.5	C29—C58—H58A	111.3
H31A—C31—H31C	109.5	C50—C58—H58A	111.3
H31B—C31—H31C	109.5	C29—C58—H58B	111.3
C41—C32—H32A	109.5	C50—C58—H58B	111.3
C41—C32—H32B	109.5	H58A—C58—H58B	109.2
H32A—C32—H32B	109.5	C52—C59—C25	101.6 (2)
C41—C32—H32C	109.5	C52—C59—H59A	111.4
H32A—C32—H32C	109.5	C25—C59—H59A	111.4
H32B—C32—H32C	109.5	C52—C59—H59B	111.4
C24—C33—H33A	109.5	C25—C59—H59B	111.4
C24—C33—H33B	109.5	H59A—C59—H59B	109.3
H33A—C33—H33B	109.5	C50—O4—C51	109.0 (2)
C24—C33—H33C	109.5	C52—O5—C53	108.96 (18)
H33A—C33—H33C	109.5	C56—O6—C46	109.45 (19)
H33B—C33—H33C	109.5	C28—O7—C35	108.6 (2)
C27—C34—C35	105.3 (3)		
			
C9—C1—C2—O1	176.94 (18)	C20—C17—C24—C26	68.6 (3)
C8—C1—C2—O1	−7.7 (3)	C12—C17—C24—C57	11.9 (3)
C9—C1—C2—C6	−1.2 (3)	C20—C17—C24—C57	−170.8 (2)
C8—C1—C2—C6	174.14 (19)	C12—C17—C24—C33	130.8 (3)
C9—C1—C2—Mg1^i^	141.50 (17)	C20—C17—C24—C33	−51.8 (3)
C8—C1—C2—Mg1^i^	−43.1 (2)	C34—C27—C28—O7	−34.5 (4)
O2^i^—C4—C5—C10	171.5 (2)	C28—C27—C34—C35	28.2 (4)
C19—C4—C5—C10	−6.5 (3)	C27—C34—C35—O7	−12.1 (4)
O2^i^—C4—C5—C8	−0.8 (3)	C23—C22—C41—C32	−104.3 (3)
C19—C4—C5—C8	−178.75 (19)	C10—C22—C41—C32	72.5 (3)
O1—C2—C6—C14	−177.94 (19)	C23—C22—C41—C31	17.8 (4)
C1—C2—C6—C14	0.2 (3)	C10—C22—C41—C31	−165.4 (3)
Mg1^i^—C2—C6—C14	−131.29 (18)	C23—C22—C41—C30	136.7 (3)
O1—C2—C6—C11	3.4 (3)	C10—C22—C41—C30	−46.6 (3)
C1—C2—C6—C11	−178.4 (2)	C58—C29—C51—O4	27.8 (3)
Mg1^i^—C2—C6—C11	50.1 (3)	C59—C25—C53—O5	−18.2 (3)
C12—C3—C7—O3	169.8 (2)	O6—C46—C54—C55	26.9 (3)
C8—C3—C7—O3	−7.3 (3)	C46—C54—C55—C56	−37.5 (3)
C12—C3—C7—C16	−9.0 (3)	C54—C55—C56—O6	35.2 (3)
C8—C3—C7—C16	173.85 (19)	C51—C29—C58—C50	−37.0 (3)
C12—C3—C8—C1	−80.2 (2)	O4—C50—C58—C29	33.7 (3)
C7—C3—C8—C1	96.8 (2)	O5—C52—C59—C25	−36.9 (3)
C12—C3—C8—C5	53.6 (3)	C53—C25—C59—C52	33.5 (3)
C7—C3—C8—C5	−129.4 (2)	C6—C2—O1—Mg1	−125.70 (18)
C9—C1—C8—C3	68.1 (2)	C1—C2—O1—Mg1	56.1 (3)
C2—C1—C8—C3	−107.2 (2)	Mg1^i^—C2—O1—Mg1	121.06 (18)
C9—C1—C8—C5	−66.0 (3)	C6—C2—O1—Mg1^i^	113.24 (18)
C2—C1—C8—C5	118.7 (2)	C1—C2—O1—Mg1^i^	−65.0 (2)
C10—C5—C8—C3	−1.2 (3)	O3—Mg1—O1—C2	−11.18 (18)
C4—C5—C8—C3	170.7 (2)	O2—Mg1—O1—C2	125.97 (16)
C10—C5—C8—C1	129.7 (2)	O1^i^—Mg1—O1—C2	−128.83 (18)
C4—C5—C8—C1	−58.4 (3)	C2^i^—Mg1—O1—C2	−146.02 (13)
C2—C1—C9—C15	1.7 (3)	Mg1^i^—Mg1—O1—C2	−128.83 (18)
C8—C1—C9—C15	−173.71 (19)	O3—Mg1—O1—Mg1^i^	117.65 (7)
C4—C5—C10—C22	5.0 (3)	O2—Mg1—O1—Mg1^i^	−105.19 (8)
C8—C5—C10—C22	176.8 (2)	O1^i^—Mg1—O1—Mg1^i^	0.0
C14—C6—C11—C39	118.9 (2)	C2^i^—Mg1—O1—Mg1^i^	−17.19 (7)
C2—C6—C11—C39	−62.5 (3)	O3—Mg1—O2—C4^i^	147.1 (2)
C14—C6—C11—C37	−0.3 (3)	O1—Mg1—O2—C4^i^	11.6 (2)
C2—C6—C11—C37	178.3 (2)	O1^i^—Mg1—O2—C4^i^	−80.7 (2)
C14—C6—C11—C40	−119.3 (2)	C2^i^—Mg1—O2—C4^i^	−90.0 (2)
C2—C6—C11—C40	59.3 (3)	Mg1^i^—Mg1—O2—C4^i^	−37.1 (2)
C7—C3—C12—C17	5.0 (3)	C3—C7—O3—Mg1	−2.4 (4)
C8—C3—C12—C17	−178.0 (2)	C16—C7—O3—Mg1	176.38 (18)
C2—C6—C14—C15	0.4 (3)	O2—Mg1—O3—C7	−166.1 (2)
C11—C6—C14—C15	179.1 (2)	O1—Mg1—O3—C7	−28.4 (3)
C1—C9—C15—C14	−1.0 (3)	O1^i^—Mg1—O3—C7	67.3 (3)
C1—C9—C15—C21	176.9 (2)	C2^i^—Mg1—O3—C7	96.4 (3)
C6—C14—C15—C9	−0.1 (3)	Mg1^i^—Mg1—O3—C7	18.3 (3)
C6—C14—C15—C21	−178.1 (2)	C58—C50—O4—C51	−16.7 (3)
O3—C7—C16—C20	−172.68 (19)	C58—C50—O4—Li1	139.7 (2)
C3—C7—C16—C20	6.2 (3)	C29—C51—O4—C50	−7.0 (3)
O3—C7—C16—C13	5.2 (3)	C29—C51—O4—Li1	−162.6 (2)
C3—C7—C16—C13	−175.93 (19)	O6—Li1—O4—C50	−79.6 (3)
C36A—C13—C16—C20	−130.1 (2)	O5—Li1—O4—C50	157.2 (2)
C49—C13—C16—C20	108.8 (2)	O7—Li1—O4—C50	38.8 (3)
C38—C13—C16—C20	−9.7 (3)	O6—Li1—O4—C51	73.1 (3)
C36A—C13—C16—C7	52.1 (3)	O5—Li1—O4—C51	−50.1 (3)
C49—C13—C16—C7	−69.0 (3)	O7—Li1—O4—C51	−168.6 (2)
C38—C13—C16—C7	172.5 (2)	C59—C52—O5—C53	26.8 (3)
C3—C12—C17—C20	2.0 (3)	C59—C52—O5—Li1	−135.9 (2)
C3—C12—C17—C24	179.5 (2)	C25—C53—O5—C52	−5.3 (3)
O2^i^—C4—C19—C23	−174.1 (2)	C25—C53—O5—Li1	155.0 (3)
C5—C4—C19—C23	3.9 (3)	O6—Li1—O5—C52	−147.6 (2)
O2^i^—C4—C19—C18	2.0 (3)	O4—Li1—O5—C52	−29.1 (3)
C5—C4—C19—C18	−180.0 (2)	O7—Li1—O5—C52	91.9 (3)
C43—C18—C19—C23	109.6 (2)	O6—Li1—O5—C53	53.8 (4)
C42—C18—C19—C23	−129.3 (2)	O4—Li1—O5—C53	172.2 (2)
C48—C18—C19—C23	−9.4 (3)	O7—Li1—O5—C53	−66.8 (3)
C43—C18—C19—C4	−66.4 (3)	C55—C56—O6—C46	−18.9 (3)
C42—C18—C19—C4	54.7 (3)	C55—C56—O6—Li1	172.0 (3)
C48—C18—C19—C4	174.6 (2)	C54—C46—O6—C56	−5.4 (3)
C7—C16—C20—C17	1.0 (3)	C54—C46—O6—Li1	164.0 (2)
C13—C16—C20—C17	−176.9 (2)	O5—Li1—O6—C56	166.7 (2)
C12—C17—C20—C16	−5.1 (3)	O4—Li1—O6—C56	47.1 (3)
C24—C17—C20—C16	177.3 (2)	O7—Li1—O6—C56	−73.3 (3)
C9—C15—C21—C47	−4.5 (3)	O5—Li1—O6—C46	−0.9 (4)
C14—C15—C21—C47	173.5 (2)	O4—Li1—O6—C46	−120.5 (2)
C9—C15—C21—C44I	−124.3 (2)	O7—Li1—O6—C46	119.1 (2)
C14—C15—C21—C44I	53.6 (3)	C27—C28—O7—C35	27.6 (3)
C9—C15—C21—C45	117.3 (2)	C27—C28—O7—Li1	−170.7 (3)
C14—C15—C21—C45	−64.8 (3)	C34—C35—O7—C28	−10.4 (4)
C5—C10—C22—C23	−0.7 (4)	C34—C35—O7—Li1	−172.0 (3)
C5—C10—C22—C41	−177.6 (2)	O6—Li1—O7—C28	−124.5 (3)
C10—C22—C23—C19	−2.1 (4)	O5—Li1—O7—C28	−0.5 (3)
C41—C22—C23—C19	174.8 (2)	O4—Li1—O7—C28	118.4 (3)
C4—C19—C23—C22	0.4 (4)	O6—Li1—O7—C35	34.3 (3)
C18—C19—C23—C22	−175.7 (2)	O5—Li1—O7—C35	158.3 (2)
C12—C17—C24—C26	−108.8 (3)	O4—Li1—O7—C35	−82.7 (3)

Symmetry code: (i) −*x*+1, −*y*+2, −*z*+1.

### Hydrogen-bond geometry (Å, º)

**Table:**

*D*—H···*A*	*D*—H	H···*A*	*D*···*A*	*D*—H···*A*
C8—H8···O1	0.98	2.33	2.867 (2)	114
C8—H8···O3	0.98	2.37	2.864 (2)	110
C36*A*—H36*C*···O3	0.96	2.30	2.935 (3)	123
C39—H39*C*···O1	0.96	2.41	3.030 (3)	122
C40—H40*C*···O1	0.96	2.40	3.025 (3)	122
C42—H42*B*···O2^i^	0.96	2.28	2.953 (3)	126
C43—H43*B*···O2^i^	0.96	2.48	3.091 (3)	122
C49—H49*A*···O3	0.96	2.47	3.094 (3)	122

Symmetry code: (i) −*x*+1, −*y*+2, −*z*+1.
